# Resolving Leukemia Heterogeneity and Lineage Aberrations with HematoMap

**DOI:** 10.1093/gpbjnl/qzaf005

**Published:** 2025-02-13

**Authors:** Yuting Dai, Wen Ouyang, Wen Jin, Fan Zhang, Wenyan Cheng, Jianfeng Li, Shuo He, Junqi Zong, Shijia Cao, Chenxin Zhou, Junchen Luo, Gang Lv, Jinyan Huang, Hai Fang, Xiaojian Sun, Kankan Wang, Saijuan Chen

**Affiliations:** Shanghai Institute of Hematology, State Key Laboratory of Medical Genomics, National Research Center for Translational Medicine at Shanghai, Ruijin Hospital, Shanghai Jiao Tong University School of Medicine, Shanghai 200025, China; Shanghai Institute of Hematology, State Key Laboratory of Medical Genomics, National Research Center for Translational Medicine at Shanghai, Ruijin Hospital, Shanghai Jiao Tong University School of Medicine, Shanghai 200025, China; Shanghai Institute of Hematology, State Key Laboratory of Medical Genomics, National Research Center for Translational Medicine at Shanghai, Ruijin Hospital, Shanghai Jiao Tong University School of Medicine, Shanghai 200025, China; Sino-French Research Center for Life Sciences and Genomics, Ruijin Hospital, Shanghai Jiao Tong University School of Medicine, Shanghai 200025, China; Shanghai Institute of Hematology, State Key Laboratory of Medical Genomics, National Research Center for Translational Medicine at Shanghai, Ruijin Hospital, Shanghai Jiao Tong University School of Medicine, Shanghai 200025, China; Shanghai Institute of Hematology, State Key Laboratory of Medical Genomics, National Research Center for Translational Medicine at Shanghai, Ruijin Hospital, Shanghai Jiao Tong University School of Medicine, Shanghai 200025, China; Shanghai Institute of Hematology, State Key Laboratory of Medical Genomics, National Research Center for Translational Medicine at Shanghai, Ruijin Hospital, Shanghai Jiao Tong University School of Medicine, Shanghai 200025, China; Shanghai Jiao Tong University School of Medicine, Shanghai 200025, China; Shanghai Jiao Tong University School of Medicine, Shanghai 200025, China; Shanghai Jiao Tong University School of Medicine, Shanghai 200025, China; Shanghai Jiao Tong University School of Medicine, Shanghai 200025, China; School of Life Sciences and Biotechnology, Shanghai Jiao Tong University, Shanghai 200240, China; Shanghai Institute of Hematology, State Key Laboratory of Medical Genomics, National Research Center for Translational Medicine at Shanghai, Ruijin Hospital, Shanghai Jiao Tong University School of Medicine, Shanghai 200025, China; Biomedical Big Data Center, the First Affiliated Hospital, Zhejiang University School of Medicine, Hangzhou 310003, China; Shanghai Institute of Hematology, State Key Laboratory of Medical Genomics, National Research Center for Translational Medicine at Shanghai, Ruijin Hospital, Shanghai Jiao Tong University School of Medicine, Shanghai 200025, China; Shanghai Institute of Hematology, State Key Laboratory of Medical Genomics, National Research Center for Translational Medicine at Shanghai, Ruijin Hospital, Shanghai Jiao Tong University School of Medicine, Shanghai 200025, China; Shanghai Institute of Hematology, State Key Laboratory of Medical Genomics, National Research Center for Translational Medicine at Shanghai, Ruijin Hospital, Shanghai Jiao Tong University School of Medicine, Shanghai 200025, China; Sino-French Research Center for Life Sciences and Genomics, Ruijin Hospital, Shanghai Jiao Tong University School of Medicine, Shanghai 200025, China; Shanghai Institute of Hematology, State Key Laboratory of Medical Genomics, National Research Center for Translational Medicine at Shanghai, Ruijin Hospital, Shanghai Jiao Tong University School of Medicine, Shanghai 200025, China

**Keywords:** Acute leukemia, Single-cell RNA sequencing, Hematopoietic hierarchy, Lineage aberration, Bioinformatics

## Abstract

Precise mapping of leukemic cells onto the known hematopoietic hierarchy is important for understanding the cell-of-origin and mechanisms underlying disease initiation and development. However, this task remains challenging because of the high interpatient and intrapatient heterogeneity of leukemia cell clones as well as the differences that exist between leukemic and normal hematopoietic cells. Using single-cell RNA sequencing (scRNA-seq) data with a curated clustering approach, we constructed a comprehensive reference hierarchy of normal hematopoiesis. This reference hierarchy was accomplished through multistep clustering and annotating over 100,000 bone marrow mononuclear cells derived from 25 healthy donors. We further employed the cosine distance algorithm to develop a likelihood score to determine the similarities of leukemic cells to their putative normal counterparts. Using our scoring strategies, we mapped the cells of acute myeloid leukemia (AML) and B cell precursor acute lymphoblastic leukemia (BCP-ALL) samples to their corresponding counterparts. The reference hierarchy also facilitated bulk RNA sequencing (RNA-seq) analysis, enabling the development of a least absolute shrinkage and selection operator (LASSO) score model to reveal subtle differences in lineage aberrancy within AML or BCP-ALL patients. To facilitate interpretation and application, we established an R-based package (HematoMap) that offers a fast, convenient, and user-friendly tool for identifying and visualizing lineage aberrations in leukemia from scRNA-seq and bulk RNA-seq data. Our tool provides curated resources and data analytics for understanding leukemogenesis, with the potential to enhance leukemia risk stratification and personalized treatments. The HematoMap is available at https://github.com/NRCTM-bioinfo/HematoMap.

## Graphical abstract

**Graphical Abstract:**
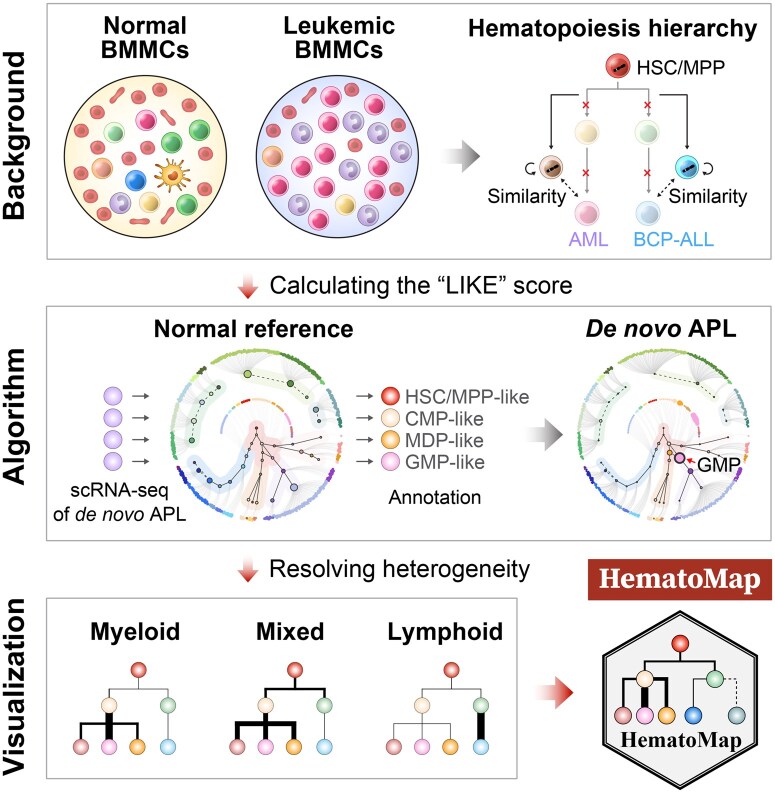

## Introduction

Acute leukemia is a malignant hematologic disease characterized by the presence of ≥ 20% immature cells derived from the bone marrow (BM), also known as leukemic blasts [1,2]. It can be classified into three broad types on the basis of the aberrant lineages involved: acute myeloid leukemia (AML), acute lymphocytic leukemia (ALL) [with 80% of patients having B cell precursor ALL (BCP-ALL)], and ambiguous lineages [including mixed phenotype acute leukemia (MPAL)] [3–5]. Understanding the aberration of leukemic blasts, particularly the cell-of-origin or leukemia-initiating cells, can aid in deeply dissecting the underlying mechanisms of leukemogenesis. Given the high degree of interpatient and intrapatient heterogeneity in morphological features, genetic alterations, and transcriptional profiles [6], characterizing the major cellular composition and hierarchy of abnormal hematopoietic cell subgroups in each patient, including changes during therapy, is increasingly important.

As a primary site for hematopoiesis, the BM comprises various hematopoietic lineages that involve hematopoietic stem/progenitor cells (HSPCs), myeloid cells [granulocytes, monocytes, dendritic cells (DCs), erythrocytes, and megakaryocytes], and lymphoid cells [B precursor cells, immature/mature B cells, and T/natural killer (NK) cells] [7–11]. Cell-type heterogeneity in human BM has been characterized recently at single-cell resolution [12–14], largely expanding our knowledge of the cellular hierarchy. The classical model of leukemogenesis views the cellular hierarchy as being built up by driver-related genetic alterations, in an attempt to seek explanations for the onset and development of aberrant leukemic cell differentiation/maturation [15–17]. Applications of single-cell RNA sequencing (scRNA-seq) and bulk RNA sequencing (RNA-seq) technologies in healthy and leukemic BM research have provided cost-effective means to understand potential leukemogenesis [18,19]. Moreover, the use of bulk RNA-seq data has greatly improved molecular subtyping and classification [20–22]. For example, a recent study associating cell differentiation stages with different AML subgroups supported the 2022 World Health Organization (WHO) classification of AML [20,23]. Here, we aim to maximize the informativeness of scRNA-seq and bulk RNA-seq data to reveal the interpatient and intrapatient cellular heterogeneity found in acute leukemia.

Hematologic malignancies differ from solid tumors partly because of the existence of lineage promiscuity, which requires highly specific cellular annotation methods. For example, inferring cell-type aberrancy by projecting leukemic blasts onto a normal cellular hierarchy has proven to be effective, allowing AML leukemic cells to be annotated as hematopoietic stem cell (HSC)-like, progenitor-like, granulocyte–monocyte progenitor (GMP)-like, monocyte-like, and conventional dendritic cell (cDC)-like [18]. Thus, there is a need to build expert-curated reference databases by integrating normal BM mononuclear cells (BMMCs) tailored to infer lineage relevance. Likewise, visually intuitive methods are needed to straightforwardly monitor cellular composition changes and lineage aberrancy during leukemogenesis in a straightforward manner.

Here, we present HematoMap, an R package designed to annotate leukemic BMMCs, infer lineage aberrancy, and visualize leukemogenesis beyond the confinement of normal hematopoietic hierarchy. The tools include well-curated datasets (both publicly available and in-house) of normal and leukemic BMMCs, totaling > 100 scRNA-seq samples and > 2000 bulk RNA-seq samples (Table S1). Through reanalysis and reannotation of the normal BMMCs, we constructed a hierarchy-based reference and developed a series of algorithms to calculate the similarities between leukemic blasts and normal hematopoietic cell types, enabling aberrant lineage inferences. Furthermore, a least absolute shrinkage and selection operator (LASSO) score model was established for bulk RNA-seq applications. Finally, all curated resources and facilitated algorithms/models were packaged into HematoMap. Using aberrant lineage inference and visualization, HematoMap provides valuable resources and analysis/visualization methods to gain a deeper understanding of the cell-of-origin in BCP-ALL and AML.

## Results

### Establishing a hierarchy-based cell reference atlas using the scRNA-seq data of normal BMMCs

**Figure 1:**
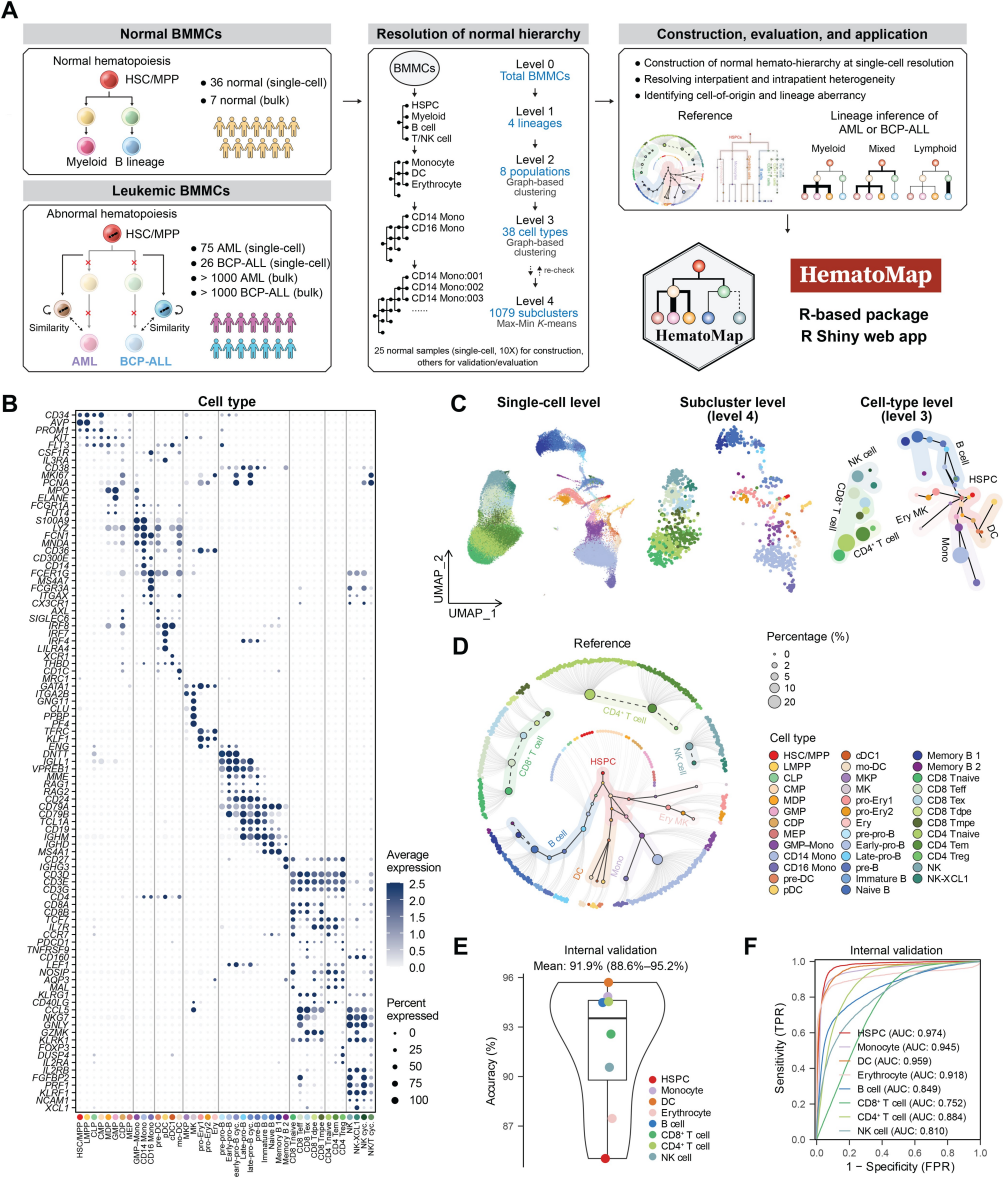
Workflow overview for resolving cell types in normal human BMMCs based on the well-established hematopoietic hierarchy **A**. Overview of the study design. The left panel shows a schematic representation of normal (upper) and abnormal (lower, the origin of acute leukemia) hematopoiesis. The bone marrow microenvironment is a cellular system consisting of various cell lineages, including immature cells such as HSPCs and mature cells such as monocytes. In this study, we first used 25 scRNA-seq datasets of normal BMMCs (10X Genomics) to perform hierarchy-based annotation. To support this application, we constructed a normal reference of BMMCs to infer the aberrancy of leukemic blasts and altered differentiation lineages on BCP-ALL and AML scRNA-seq and bulk RNA-seq. All of these can be performed in our newly developed R-based package HematoMap to support further leukemic research. **B**. Dot plot of markers of different cell types (38 main cell types and 4 cycling cell types) in normal BMMCs. **C**. Characterization of the cell types identified from the 25 normal BMMC samples. UMAP was used for dimensionality reduction and visualization. For the left panel, dots represent cells (the single-cell level). For the middle panel, dots represent subclusters (the subcluster level). For the right panel, dots represent cell types (the cell-type level). The different colors indicate different cell types. The coordinates of subclusters/cell types were calculated using the mean value of the included cells. For visualization at the cell-type level, the continuous differentiation was labeled with a black line. HSCs/MPPs are at the differentiation initiation site. **D**. Hierarchy-based visualization of normal hematopoiesis. The inner circle shows the 38 major cell types identified in normal BMMCs, with the subclusters of each cell type being arranged around the circle’s edge. The solid lines represent continuous differentiation (HSPCs, monocytes, erythrocytes, and B cell lineage), the dashed lines represent recruited mature cells from other tissues (T/NK cells and some memory B cells), and the gray lines represent links between each cell type and its subclusters. The cell types, represented as circles, are color-coded. **E**. Annotation accuracy comparing annotation using SingleR (the subclusters obtained from the 25 normal BMMC samples were input as the reference) and manual annotation using classical markers. The annotation accuracy of each cell population was visualized using a box plot, with the mean value and 95% CI illustrated. **F**. ROC curve and AUC analyses revealed high sensitivity and specificity and favorable performance when the annotation reference constructed from the 25 normal BMMC samples was used. HSPC, hematopoietic stem/progenitor cell; scRNA-seq, single-cell RNA sequencing; BMMC, bone marrow mononuclear cell; BCP-ALL, B cell precursor acute lymphoblastic leukemia; AML, acute myeloid leukemia; RNA-seq, RNA sequencing; UMAP, Uniform Manifold Approximation and Projection; ROC, receiver operating characteristic; AUC, area under the curve; CI, confidence interval; FPR, false positive rate; TPR, true positive rate; HSC/MPP, hematopoietic stem cell/multipotent progenitor; LMPP, lymphoid-primed multipotent progenitor; CLP, common lymphoid progenitor; CMP, common myeloid progenitor; MDP, monocyte–dendritic cell progenitor; GMP, granulocyte–monocyte progenitor; CDP, common dendritic cell progenitor; MEP, megakaryocyte–erythrocyte progenitor; Mono, monocyte; GMP–Mono, GMP–monocyte; CD14 Mono, classical (CD14^+^CD16^−^) monocyte; CD16 Mono, non-classical (CD14^−^CD16^+^) monocyte; DC, dendritic cell; pre-DC, dendritic cell precursor; pDC, plasmacytoid dendritic cell; cDC1, conventional dendritic cell 1; mo-DC, monocyte-derived dendritic cell; MKP, megakaryocyte progenitor; MK, megakaryocyte; pro-Ery1, proerythroblast 1; pro-Ery2, proerythroblast 2; Ery, erythroblast; pre-pro-B, B cell progenitor precursor; Early-pro-B, early B cell progenitor; early-pro-B cyc., early cycling B cell progenitor; Late-pro-B, late B cell progenitor; late-pro-B cyc., late cycling B cell progenitor; pre-B, B cell precursor; Immature B, immature B cell; Naive B, naive B cell; Memory B 1, memory B cell 1; Memory B 2, memory B cell 2; CD8 Tnaive, naive CD8^+^ T cell; CD8 Tdpe, KLRG1^+^IL7R^+^ double-positive effector CD8^+^ T cell; CD8 Tmpe, memory precursor effector CD8^+^ T cell; CD8 Teff, effector CD8^+^ T cell; CD8 Tex, exhausted CD8^+^ T cell; CD4 Tnaive, naive CD4^+^ T cell; CD4 Tem, effector memory CD4^+^ T cell; CD4 Treg, regulatory T cell; NK, natural killer cell; NK-XCL1, XCL1^+^ natural killer cell; NK cyc., cycling natural killer cell; NK/T cyc., cycling natural killer/T cell.

We performed a hierarchy-based cell-type annotation by combining the scRNA-seq (10X Genomics) data of normal BMMCs from scRNA-seq dataset 1 (scDS1) [24], scDS2 [25], and scDS3 [13] (Table S1). A total of 25 normal BMMC samples (23 adults and 2 children, scRNA-seq via 10X Genomics) were used to construct the reference atlas, whereas the other BMMCs (samples taken from the same scDS3 donors at different time points, or scRNA-seq via non-10X Genomics platforms) were used for independent validation. After batch effects were corrected via Harmony [26], a total of 103,871 BMMCs were obtained to construct the normal hematopoiesis hierarchy. We identified four general lineages (HSPCs, myeloid cells, B cells and T/NK cells, Level 1) according to well-established cell markers (the clustering procedures are described in the Materials and methods) and markers for cell-type annotation, with the related references listed in Table S2 (**Figure 1**A) [14]. On the basis of the four lineages, eight major cell populations were further identified via graph-based clustering following the Seurat standard workflow [27]; these cell populations included HSPCs, monocytes, DCs, erythrocytes, B cells, CD8^+^ T cells, CD4^+^ T cells, and NK cells (Figure S1A), each characterized by distinct markers (Figure S1B). Subsequently, the cells from each population were extracted and subjected to an independent graph-based cell clustering using Seurat [27] and an annotation process (Figure 1A). To achieve more precise subclustering of normal BMMCs, we also employed a Max-Min *K*-means algorithm to generate subclusters (Level 4) with the desired median number (100) of cells per subcluster (Figure S1C). The cell types were annotated according to well-defined cell markers, and a total of 38 cell types (Level 3) were annotated (Figure 1B and D). The detailed sample characteristics and cellular composition information for the normal BMMC samples are listed in Table S3.

To better illustrate subtle differences in intra-cell-type heterogeneity and discover lineage branching, we performed diffusion pseudotime (DPT) [28] using the 1079 preidentified subclusters (Figure S1D). Temporal cellular ordering, together with prior knowledge in hematology, enabled the construction of the hierarchy-based hematopoiesis (Figure 1C, Figure S1E), forming an expression-based cell-type annotation reference. We used a circular layout for visualizing hierarchy-based hematopoiesis, which displays the 38 major cell types and their subclusters (Figure 1D, Figure S1F). In this layout, HSC/multipotent progenitor (MPP) sits at the initiation site of hematopoiesis from which four continuously differentiated lineages (erythrocytes, monocytes, DCs, and B cells) extend. For T cell development, common lymphoid progenitors (CLPs) migrate from the BM to the thymus, where they mature and differentiate into different types of T/NK cells, such as CD8^+^ T, CD4^+^ T, and NK cells [29]. Thus, in human BM, mature T/NK cells are recruited from peripheral blood and participate in the immune microenvironment constitution. Therefore, we used solid lines to indicate continuous differentiation and dashed lines to denote cell recruitment from other tissues (Figure 1D).

We then internally evaluated the robustness of the subcluster expression profile using different sample sizes and cell numbers. By calculating the correlation coefficient between the expression profiles of the reference subclusters and the downsampled subclusters, we found that the correlations increased when the sample size exceeded 12 or the cell number exceeded 50,000 (Figure S1G and H). This finding highlights the reliability and robustness of using 25 normal BMMC samples as the reference. We also evaluated the reliability of subcluster annotation based on 1079 subclusters annotated via an unbiased tool, SingleR [30], as the third-party evaluation method for self-validation. The median accuracy of the 25 normal BMMC samples was 91.9% [95% confidence interval (CI) = 88.6%–95.2%] (Figure 1E), and the area under the curve (AUC) was > 0.9 for cells in myeloid lineages and ∼ 0.8 for those in lymphoid lineages (Figure 1F). For external validation, we also used other scRNA-seq data of normal BMMCs in terms of recovering the classical markers (Table S1), with mean accuracies of 92.3% (95% CI = 86.5%–98.1%) for 10X Genomics (other normal BMMCs in scDS3) (Figure S1I and J) and 80.4% (95% CI = 70.7%–90.0%) for non-10X Genomics (scDS4 [18] and scDS5 [19]) (Figure S1K and L). These results provided evidence supporting the informativeness of subclusters in capturing representative features of each cell type and highlighted the utility of annotating healthy BMMCs in near future research. The expression-based cell-type annotation reference was packaged into our developed R-based package, HematoMap, which provides a convenient tool for BMMC annotation.

### Inferring the leukemic lineage aberrancy via scRNA-seq

**Figure 2:**
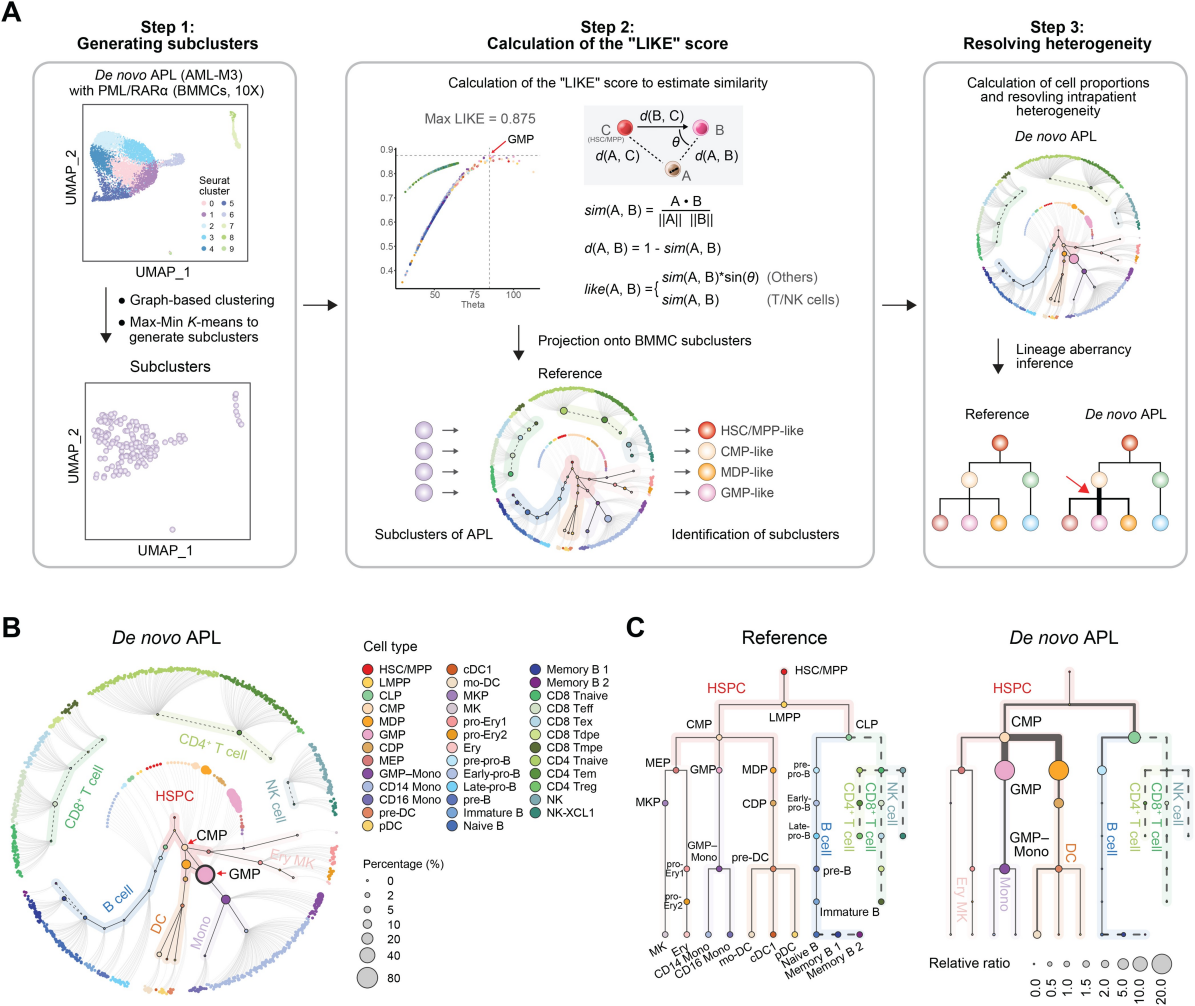
Estimation of similarities between leukemic blasts and lineage aberrancy inference from scRNA-seq data in a *de novo* APL patient **A**. Illustration of the algorithm used to calculate the LIKE score. The distance *d*() between subclusters is defined using the cosine similarity distance, which acts as a metric to compare gene expression profile vectors between leukemia-associated subclusters (matrix A) and reference subclusters consisting of normal BMMCs (matrix B) and HSC/MPP subclusters (matrix C). The LIKE score for each leukemia-related subcluster is derived from these cosine similarity calculations, which quantify the extent to which each leukemia subcluster resembles the gene expression patterns of the normal BMMC subclusters. **B**. Hierarchy-based visualization of the cellular composition of *de novo* APL. The inner circle shows the 38 major cell types identified in normal BMMCs, and the subclusters in each cell type are arranged around the edge of the circle. The solid lines represent continuous differentiation (HSPCs, monocytes, DCs, erythrocytes, and B cell lineage), the dotted lines represent recruited mature cells from other tissues (T/NK cells and some memory B cells), and the gray lines represent links between each cell type and its subclusters. Dots are color-coded by cell type. **C**. Tree plot visualization of the lineages of normal BMMCs (left) and APL BMMCs (right). The cell types, represented as circles, are color-coded, with HSC/MPP sitting at the initiation site of the hierarchy. The size of the circle represents the relative ratio of cell proportions in leukemic (*e.g*., APL)/normal (reference) BMMCs. A relative ratio > 1 represents proliferation, whereas a ratio < 1 represents suppression. The aberrant lineages in APL are indicated by thicker lines. The solid lines depict continuous differentiation processes within bone marrow hematopoiesis, and the dashed lines denote cells recruited into the bone marrow from other tissues, such as T and NK cells. APL, acute promyelocytic leukemia; LIKE score, likelihood score.

The hematopoietic hierarchy in leukemia settings differs from that under physiologic conditions. However, despite the aberrant hematopoietic cell self-renewal and differentiation, leukemic blasts might still exhibit some similarities with their normal counterparts. To address this issue, we developed an efficient approach to estimate the similarity between leukemic blasts and normal blood cells in BMMCs, taking a patient with *PML::RARA* AML [also known as acute promyelocytic leukemia (APL)] as an example (**Figure 2**A). Briefly, we first performed a routine analysis process on the scRNA-seq data of the BMMCs from this patient following the Seurat standard workflow [27]. Subclusters were then generated, followed by estimation of the similarities between the normal and APL subclusters (Step 1 in Figure 2A). A likelihood score (the “LIKE” score) of the APL subclusters was subsequently calculated by the cosine distance from the APL subcluster to the differentiation processes initiating from the HSC/MPP (Step 2 in Figure 2A, Figure S2A). The cell types of the APL subclusters were determined by the maximum LIKE score (Step 2 in Figure 2A, Figure S2A). As a result, each APL subcluster was closely mapped onto a subcluster mimicking normal counterparts, such as HSC/MPP-like, common myeloid progenitor (CMP)-like, or GMP-like (Step 2 in Figure 2A). This allowed us to infer aberrant lineages during leukemogenesis by comparing changes in cellular composition with the normal hierarchy (Step 3 in Figure 2A).

Additionally, when this APL patient was used as an example, we reannotated the subclusters of the APL BMMCs by calculating the LIKE score (Figure S2A). Leukemic blasts in APL patients were previously reported to share similar GMP expression signatures [18,20,31]. Using the LIKE score, the major cell population with the highest percentages in the APL patients was identified as GMP-like (Figure 2B). A comparison of the expression levels of classical GMP markers revealed high similarities between GMP-like cells and normal GMP cells, facilitating the analysis of dysregulated pathways between APL blasts and corresponding normal cell populations [32] (Figure S2B and C). Although APL blasts could be identified as GMP-like, their general score in APL patients was significantly lower than that in normal BMMCs, indicating that leukemic blasts in APL patients are unable to perform their normal biological functions (Figure S2D). Considering the cellular composition changes that occurred in leukemic hematopoiesis compared with normal hematopoiesis, the aberrant lineages in APL patients could be inferred and visualized in a tree-shaped hierarchy (Figure 2C). Additionally, we conducted a self-verification to evaluate the performance of the LIKE score on normal BMMCs and found that it exhibited comparable performance to that of SingleR (Figure S2E). Early-stage lineage aberrations were not observed in two additional datasets for normal BMMCs from the study by Weng et al. [33] (Figure S2F and G), indicating that the aberrations identified in leukemic samples were specific to leukemogenesis and not artifacts in healthy samples.

### Discovering cellular composition changes and lineage aberrations in AML and BCP-ALL scRNA-seq cohorts

**Figure 3:**
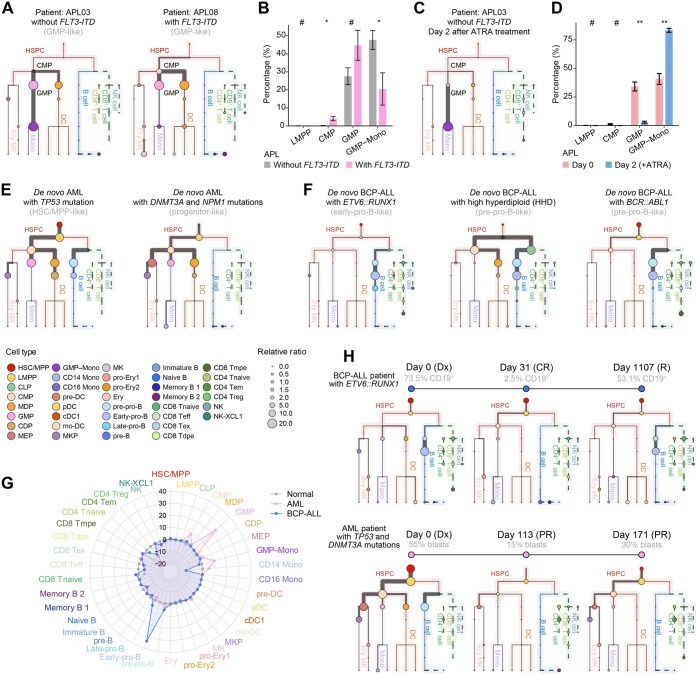
Inference of leukemic aberrant lineages in the AML and BCP-ALL scRNA-seq cohorts **A**. Tree plots displaying the aberrant lineages in two *de novo* APL patients (AML with *PML::RARA* fusion). The first patient (APL03) did not carry the *FLT3-ITD* mutation, whereas the second patient (APL08) did. The circle size represents the ratio of the total number of cells to the total number of normal BMMCs (the relative ratio). The thickness of the line indicates the proportion of cells within that lineage. **B**. Box plot revealing the estimated percentages of the cell populations in 16 APL patients (*n* = 11 for APL patients without the *FLT3-ITD* mutation and *n* = 5 for APL patients with the *FLT3-ITD* mutation). The percentages of cells were estimated via HematoMap. *P* values were calculated via the Mann–Whitney U test (#, *P* ≥ 0.05; *, *P* < 0.05; **, *P* < 0.01). **C**. Tree plot of the APL patient (APL03) after two days of ATRA treatment. **D**. Box plot revealing the estimated percentages of the cell populations in 3 APL patients on Day 0 and after two days of ATRA treatment (Day 2). *P* values were calculated using the paired Mann–Whitney U test (#, *P* ≥ 0.05; *, *P* < 0.05; **, *P* < 0.01). **E**. Tree plots displaying the aberrant lineages in the two *de novo* AML patients from scDS4. The first patient, harboring the *TP53* mutation, was validated to be HSC-like via flow cytometry in a previous study (Patient AML916). *DNMT3A* and *NPM1* mutations were detected in the second AML patient from scDS4, and the patient was reported to be progenitor-like (Patient AML419). The thickness of the line indicates the proportion of cells within that lineage. **F**. Tree plots displaying the aberrant lineages of the three *de novo* BCP-ALL patients from scDS1 (scRNA-seq, 10X Genomics) and scDS2 (scRNA-seq, 10X Genomics). Aberrations in B cell lineages occurred in BCP-ALL patients with *ETV6::RUNX1* and *BCR::ABL1*, whereas in highly hyperdiploid BCP-ALL patients, aberrations occurred in both CMP and CLP cells. **G**. Radar plot of changes in the cellular composition of AML (pink line) and BCP-ALL (blue line) cells compared with normal BMMCs. **H**. Tree plots depicting changes in the aberrant lineages of two patients (one BCP-ALL patient harboring *ETV6::RUNX1* and one AML patient harboring *TP53* and *DNMT3A* mutations) during treatment. ATRA, all-*trans* retinoic acid; Dx, diagnosis; CR, complete remission; R, relapse; PR, partial remission.

Using the established workflow, we analyzed BMMCs from 16 APL patients to assess their proportions (**Figure 3**A, Figure S3A). Our previous research revealed that APL patients with *FLT3-ITD* mutations presented increased percentages of stem-like cells [32], which is consistent with the hierarchies illustrated via HematoMap (Figure 3B). Additionally, the efficacy of all-*trans* retinoic acid (ATRA) treatment in inducing the differentiation of primitive APL blasts toward more mature cells was effectively demonstrated through hierarchical illustrations, confirming the utility of our method in monitoring treatment response (Figure 3C and D, Figure S3B). In addition, we calculated the LIKE scores and performed lineage aberration inference for the AML (scDS4 and scDS5) and BCP-ALL (scDS1 and scDS2) scRNA-seq cohorts. For other *de novo* AML patients, interpatient and intrapatient heterogeneity was investigated, and the results suggested that different cells-of-origin in AML samples were accompanied by different gene fusions or mutations (Figure 3E). For the AML samples harboring a *TP53* mutation, the initiating site of lineage aberrancy started from the HSC/MPP, and both the myeloid and lymphoid lineages were altered (Figure 3E). For the progenitor-like-containing samples, the aberrations appeared mainly in the myeloid lineage, and the initiating site was at the lymphoid-primed multipotent progenitor (LMPP) stage (Figure 3E). Aberrancies in the B cell lineage were also observed in the BCP-ALL patients (Figure 3F). For the leukemia patients harboring *ETV6::RUNX1*, an aberrancy in the CLP–pre-pro-B–early-pro-B differentiation axis was observed (Figure 3F). For the two BCP-ALL patients with high hyperdiploidy and harboring *BCR::ABL1*, aberrancy occurred mainly in the B cell lineages, and the CMP was slightly affected, which was indicative of MPAL (Figure 3F).

We then compared the lineage aberrations in AML and BCP-ALL BMMCs with those in normal BMMCs via a radar plot to illustrate the cellular composition (Figure 3G). For *de novo* AML patients, the aberrations of leukemic blasts were mainly at CMP, GMP, and GMP–monocyte stages (Figure 3G). For BCP-ALL patients, the aberrations were at the pre-pro-B, early-pro-B, and late-pro-B stages (Figure 3G). Intrapatient cellular heterogeneity was investigated among the different French–American–British (FAB) subtypes by comparing the cellular composition changes among different *de novo* AML patients in scDS5 (Figure S3C). In addition, lineage aberrancy was also observed in AML and BCP-ALL patients at different treatment time points (Figure 3F and H). In two patients receiving treatment, leukemic BMMCs during complete remission were similar to normal BMMCs, but the residual leukemic blasts grew and relapsed at initial hierarchy sites similar to those identified at the time of diagnosis (Figure 3F and H).

### Applying a cell-type-based score to AML and BCP-ALL bulk RNA-seq data

**Figure 4:**
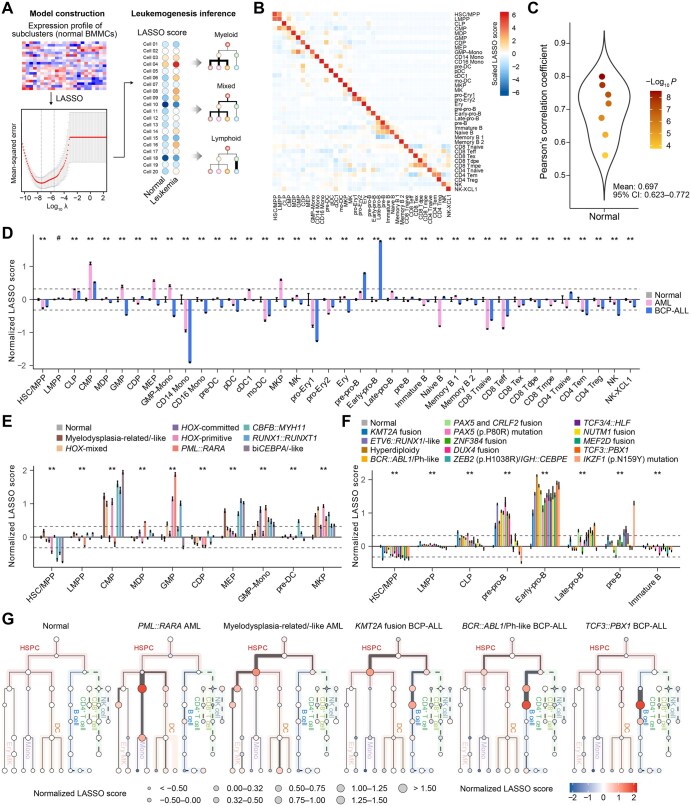
Inference of leukemic aberrant lineages in the AML and BCP-ALL bulk cohorts **A**. Overview of the construction of the LASSO-based score model. **B**. Heatmap visualization of the LASSO score for each cell type via scRNA-seq data. LASSO scores were calculated based on the mean values of scores in each subcluster and normalized by the z-score. **C**. Violin plot of self-validation using the seven available normal BMMC samples with both scRNA-seq (10X Genomics) and bulk RNA-seq data. Pearson’s correlation coefficients were calculated between the LASSO scores from the scRNA-seq and bulk RNA-seq data. Each circle represents one sample. **D**. Bar plot of normalized scores in normal, AML, and BCP-ALL BMMCs from bulk RNA-seq data. Normalization was performed by subtracting the mean values of normal samples of each cell type. *P* values were calculated via ANOVA (#, *P* ≥ 0.05; *, *P* < 0.05; **, *P* < 0.01). **E**. Bar plot of the normalized scores from normal BMMCs and AML patients in different subgroups. *P* values were calculated via ANOVA (**, *P* < 0.01). **F**. Bar plot of the normalized scores from normal BMMCs and BCP-ALL patients in different subgroups. *P* values were calculated by ANOVA (**, *P* < 0.01). **G**. Tree plots depicting inferred lineage aberrations according to normalized LASSO scores of normal BMMCs and representative subgroups of AML and BCP-ALL patients. For myelodysplasia-related/-like AML patients, the aberrations started from HSC/MPP/LMPP and mainly influenced the myeloid lineages (for most AML patients). For BCP-ALL patients with *KMT2A* fusion, both the myeloid and lymphoid lineages were affected (for MPAL). For BCP-ALL patients with *BCR::ABL1*, the aberrations started from LMPP and mainly influenced the B cell lineage (for most BCP-ALL patients). For BCP-ALL patients with *TCF3::PBX1*, the alterations were mostly observed in pre-pro-B, early-pro-B, and late-pro-B lineages. The thickness of the line indicates the proportion of cells within that lineage. LASSO, least absolute shrinkage and selection operator; ANOVA, analysis of variance; MPAL, mixed phenotype acute leukemia.

To infer lineage aberrations from bulk RNA-seq data, we constructed a LASSO-based score model to calculate cell abundances and infer leukemogenesis (**Figure 4**A). The construction was based on the mean expression profile of subclusters (see Materials and methods), and scores for the 38 cell types were established independently (Figure 4A). The coefficients of LASSO derived from the 38 normal cell types are listed in Table S4. To self-validate the model at the scRNA-seq level, we calculated the LASSO score for each cell type, with high specificity observed in normal BMMCs (Figure 4B). To further evaluate the performance of bulk RNA-seq, we used seven normal BMMC samples for which both scRNA-seq and bulk RNA-seq data (bulkDS1 [24]) were available. The correlation between the LASSO score calculated from scRNA-seq and that from bulk RNA-seq was significant (mean Pearson’s correlation coefficient = 0.697, 95% CI = 0.623–0.772) (Figure 4C). The 38 cell-type LASSO scores of the 7 normal BMMCs were then normalized by subtracting the internal average score to select the optimal cutoff to distinguish between normal and abnormal scores (Figure S4A). We then applied the LASSO score to bulk RNA-seq cohorts of AML (bulkDS2 [34], bulkDS3 [35], and bulkDS4 [20]) (Table S1) and BCP-ALL (bulkDS5 [21]) (Table S1). Patients in bulkDS4 and bulkDS5 were collected from multiple centers, and the detailed LASSO scores of the AML/BCP-ALL patients are listed in Table S4. After normalization to normal BMMCs, most AML patients were assigned higher CMP/GMP/megakaryocyte–erythrocyte progenitor (MEP)/megakaryocyte progenitor (MKP)/GMP–monocyte scores (fold change > 1.25) (Figure 4D), whereas higher pre-pro-B/early-pro-B scores were observed for patients diagnosed with BCP-ALL (fold change > 1.25) (Figure 4D).

A previously described framework by our own group [20,21] also enabled the examination of interpatient heterogeneity (Figure S4B and C). For AML patients, within the preestablished classification framework, those classified as myelodysplasia (MD)-related/-like or *HOX*-primitive/-mixed or bi*CEBPA*/-like AML patients, or those with *CBFB::MYH11* or *RUNX1::RUNX1T1*, had higher CMP scores, suggesting that the aberrancy mainly occurred at the CMP stage and affected the differentiation of monocytes, DCs, and erythrocytes (Figure 4E, Figure S4B). Patients with *PML::RARA* fusion had significantly higher GMP scores (Figure 4E, Figure S4B). The same cell-type signatures of AML were also observed in external independent cohorts of AML patients (TCGA-LAML and Beat-AML) (Figure S4D–F; Table S5). With respect to BCP-ALL, B cells in patients with *KMT2A* fusion, *ETV6::RUNX1*/-like, hyperdiploidy, *BCR::ABL1*/Ph-like, *PAX5* and *CRLF2* fusion, *PAX5* (p.P80R) mutation, *ZNF384* fusion, or *DUX4* fusion were blocked mainly at the pre-pro-B and early-pro-B stages (Figure 4F, Figure S4C). Patients with *ZEB2* (p.H1038R)/*IGH::CEBPE*, *TCF3/4::HLF*, *NUTM1* fusion, *MEF2D* fusion, *TCF3::PBX1*, or *IKZF1* (p.N159Y) mutation had higher early/late-pro-B signatures (Figure 4F, Figure S4C).

For illustration, we selected five representative lineage aberrations of AML and BCP-ALL, including two myeloid models for AML, one mixed lineage model for MPAL, and two lymphoid models for BCP-ALL (Figure 4G). Generally, for normal BMMCs, the relative ratio for each cell type was normalized to nearly 1.0, which could help us better interpret aberrations in AML and BCP-ALL (Figure 4G). Compared with those in the *PML::RARA* AML patients, leukemic cell aberrations in the MD-related/-like AML patients occurred relatively earlier, with a focus on the LMPP–CMP differentiation axis (Figure 4G). With respect to BCP-ALL, *KMT2A* fusion was previously reported to belong to MPAL [4], and the bilineage aberration was observed via tree-shaped visualization (Figure 4G). For the other two subtypes of BCP-ALL, *BCR::ABL1*/Ph-like BCP-ALL tended to be MPAL and the lineages were altered mainly on the B cell differentiation axis, whereas *TCF3::PBX1* BCP-ALL was at the early-pro-B stage (Figure 4G).

### Development and deployment of the HematoMap package

**Figure 5:**
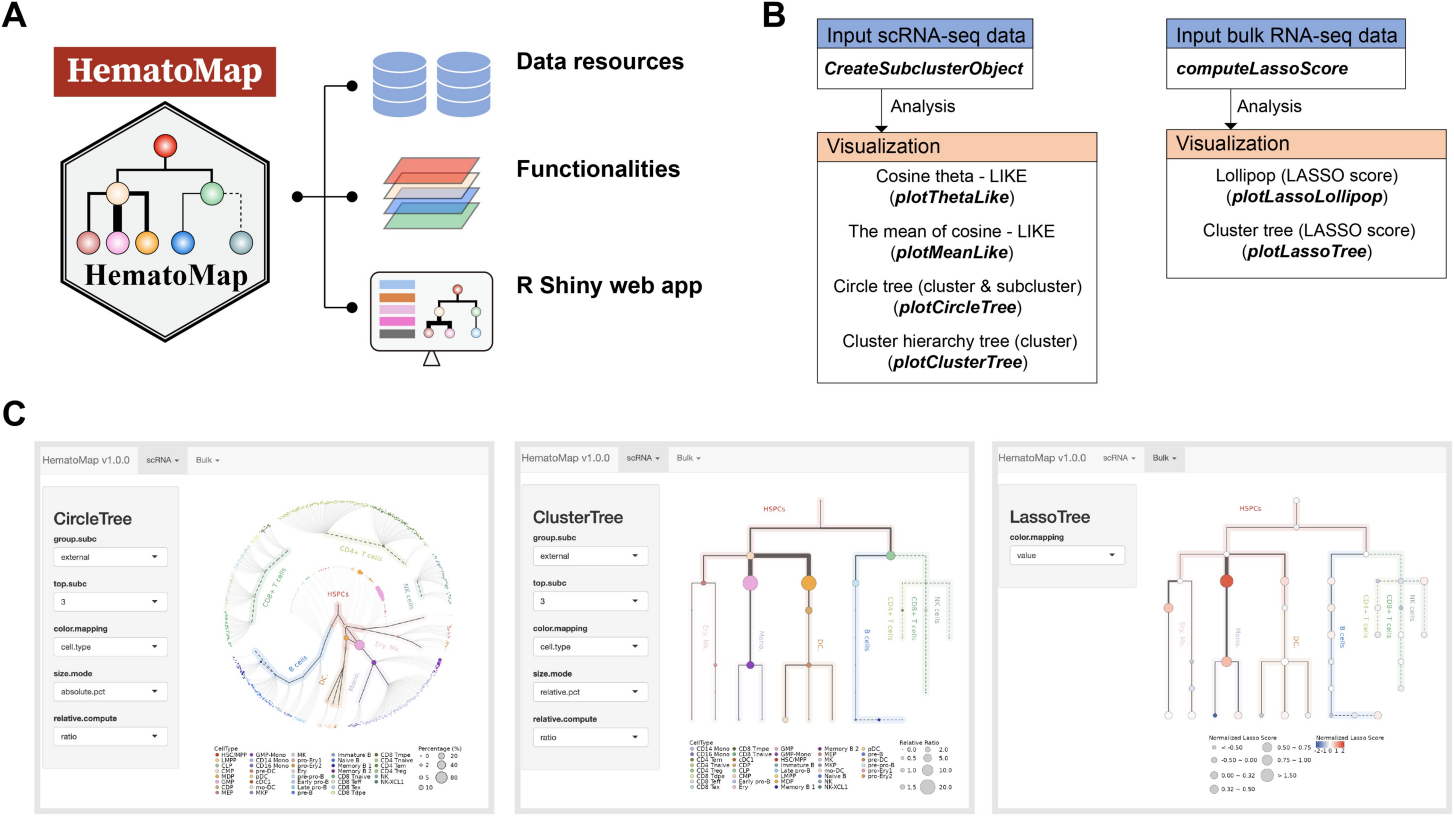
Overview of the HematoMap package design, main functionalities, and analysis workflow **A**. Structure of the HematoMap package. **B**. Overview of the main functionalities in HematoMap. The headers of boxes describe available functional modules, including input and visualization. The brief descriptions and the corresponding functions are listed in module boxes. **C**. Snapshots of applications for the visualization of scRNA-seq and bulk RNA-seq data developed using the R Shiny package. The left panel shows the tool for visualizing the circle tree of the example scRNA-seq data (taking the APL sample as an example). The middle panel shows the visualization of the cluster tree. The right panel shows the tool for mapping the LASSO score of bulk RNA-seq data to the cluster tree.

We developed an R package, HematoMap, that offers a comprehensive analysis workflow for cell-type annotation, lineage aberrancy inference, and visualization in both scRNA-seq and bulk RNA-seq applications (**Figure 5**A). Our package is built on preannotated normal BMMCs and preestablished algorithms to ensure the accuracy and reliability of the results. HematoMap can run seamlessly on several platforms, including Linux, Windows, and macOS, and provides an easy-to-use workflow for users (Figure 5B). One of the key features of HematoMap is its manually curated annotation database for normal BMMCs. This resource is particularly useful for researchers studying hematopoietic hierarchy. Moreover, we developed an R Shiny-based interactive interface that is even easier to use (Figure 5C). The main functionalities developed in HematoMap provide services directed from scRNA-seq and bulk RNA-seq data (Figure 5C). The package can generate subclusters on the basis of cell-type identification, and the cell types can be mapped onto a circular tree for visualization purposes (Figure 5C). The package also offers a tree-shaped visualization for lineage aberrancy interpretation, making it easier to identify abnormalities in the cell lineage (Figure 5C). Overall, HematoMap provides a powerful tool for the analysis of hematopoietic cell populations, with broad applications in diverse research areas.

## Discussion

Understanding the cell-of-origin and pathogenic mechanisms of acute leukemia, an aggressive hematologic malignant disease, has received much attention. A number of classical models, including the genomic [4], stem cell [36], and “multihit” [37] models, have been proposed, but they were all built on the basis of normal hematopoiesis. The accumulation of transcriptome-based profiles of acute leukemia, coupled with the integration and reanalysis of scRNA-seq and bulk RNA-seq data, has greatly improved the disease classification framework, resulting in new insights into leukemogenesis [21,38–41]. Therefore, a curated resource based on the scRNA-seq data of normal BMMCs will be useful for researchers investigating leukemogenesis. With HematoMap, we offer a resource database of normal BMMCs and a series of analyses to infer leukemogenesis from scRNA-seq/bulk RNA-seq data from AML and BCP-ALL. This package should be helpful in analyzing large AML and BCP-ALL cohorts with high performance particularly when in-house generated and external validation data are used. With a user-friendly workflow, HematoMap allows running from data input to output visualization and has the potential to support cell-of-origin exploration and therapeutic target identification for relevant leukemia settings in the future.

The interpatient and intrapatient heterogeneity in acute leukemia is complex because of the genetic alterations involved. By reclassifying transcriptome-wide subtypes of acute leukemia, we determined the similarities among the different subgroups [21]. At the molecular level, patients in the same subgroup, such as *BCR::ABL1*/Ph-like patients, might harbor different fusions/mutations but still share similar gene expression patterns and be sensitive to the same treatment. This knowledge lays a foundation for transcriptome-wide subtyping and treatment–response prediction. Conversely, through in-depth investigations via scRNA-seq, patients in the same subgroup might harbor similar cellular compositions and cells-of-origin at the phenotypic level. For deconvolution analysis of bulk RNA-seq data in hematologic malignancies, the similar expression signatures between leukemic blast cells and deviations during percentage estimation necessitate more precise methods. We chose LASSO because it offers greater sensitivity to character selection and superior speed and efficiency in analyzing gene expression data compared with well-established tools such as CIBERSORTx [42] and EcoTyper [43,44]. Different deconvolution methods are suitable for different scenarios, and combining multiple methods for application is encouraged [32]. In general, HematoMap helps deconvolute the cellular abundance levels, infer lineage involvement, illustrate changes in the cellular composition, and identify dysregulated genes relevant to leukemogenesis on the basis of scRNA-seq and bulk RNA-seq data.

There are still limitations to HematoMap. One limitation is the establishment of the LIKE score for the continuous differentiation axis, which might inflate the differences among various cell differentiation stages. Because T cell development and maturation do not occur in the BM, HematoMap cannot be used to calculate the LIKE score for T-ALL patients. Additionally, there are still limitations in capturing neutrophils in the BM with the existing technology, highlighting the need for more targeted research on neutrophil differentiation. Another limitation is the similar gene expression signatures among different normal HSPCs. Although the specificity of each gene expression signature of the cell subclusters was enhanced by the cosine distance and LIKE score, collinearity was not completely eliminated. However, with the increasing availability of multi-omics data, including proteomic and epigenomic data, HematoMap could be further expanded to encompass more resources and functionalities.

## Conclusion

This study presents a comprehensive reanalysis of scRNA-seq and bulk RNA-seq data from normal and leukemic BMMCs. Our efforts resulted in the construction of a hierarchy-based reference for normal hematopoiesis at single-cell resolution. Furthermore, we have developed the LIKE score to estimate the cellular compositions of AML and BCP-ALL leukemic BMMCs. By systematically comparing cellular composition changes with those observed in normal BMMCs, we successfully inferred lineage aberrations and organized them within the framework of the established hierarchy-based normal reference. The integration of these findings, along with curated database resources, novel algorithms, and advanced visualization tools, has culminated in the creation of HematoMap. The performance and utility of HematoMap have been demonstrated through applications using scRNA-seq and bulk RNA-seq data in large AML and BCP-ALL cohorts. We anticipate that HematoMap will be an invaluable tool for the hematology/oncology scientific community, offering capabilities for reference resource sharing, lineage aberration inference and visualization, and as illustrated in this study, transcriptomic insights into the cell-of-origin underlying the pathogenesis of AML and BCP-ALL.

## Materials and methods

### Sample and data collection

In this study, all the included datasets were collected from previous publications, as shown in Table S1, and then analyzed. For scRNA-seq data collection (scDS1–6), raw sequencing data were downloaded from the Gene Expression Omnibus (GEO) of the National Center for Biotechnology Information (NCBI, https://www.ncbi.nlm.nih.gov/geo/) [45] or the Genome Sequence Archive for Human (GSA-Human) of the National Genomics Data Center (NGDC), China National Center for Bioinformation (CNCB) (https://ngdc.cncb.ac.cn/gsa-human) [46]. The raw sequencing data of scDS1 [24] (GEO: GSE132509), scDS2 [25] (GEO: GSE130116), and scDS3 [13] (GEO: GSE120221) were downloaded and transferred into FASTQ format using the SRA Toolkit (v2.11.0). To obtain gene expression matrices from scDS1, scDS2, scDS3, and scDS6, we used Cell Ranger (10X Genomics, default settings, v6.0.2) to align the scRNA-seq FASTQ data onto the human GRCh38 reference (2020-A version). Both the Cell Ranger software and the human reference were downloaded from the 10X Genomics website (https://www.10xgenomics.com/). For samples in scDS4 [18] and scDS5 [19], the raw sequencing data were downloaded from the GEO database (GEO: GSE116256 and GSE130756, respectively) and transferred into FASTQ format using the SRA Toolkit. The expression profiles of scDS4 and scDS5 were obtained from the supplementary files in GEO. For the collection of bulk RNA-seq data, raw data from bulkDS1 were also downloaded from the GEO database (GEO: GSE120444). The expression profile of bulkDS2 (TCGA-LAML) was downloaded from the NCBI’s Genomic Data Commons (GDC, https://portal.gdc.cancer.gov/). For bulkDS3 (Beat-AML), the expression profile was obtained from the supplementary data of the original paper [35]. For the BCP-ALL bulk RNA-seq data (bulkDS4), the bulk RNA-seq and clinical datasets were collected from five significant research cohorts, including 1223 BCP-ALL cases available from our previous study [21]. For the AML bulk RNA-seq cohort (bulkDS5), the data were collected as described in our previous study [20]. For scRNA-seq of APLs, the raw sequencing data were downloaded from the GSA-Human (GSA-Human: HRA003777) [32].

### Preprocessing of scRNA-seq data

To integrate the scRNA-seq data, the gene expression profile of each sample was imported into the Seurat R package [27]. Low-quality cells (expressed genes < 300, unique counts > 30,000 or < 500, expressed mitochondrial RNA > 10%, or marked doublets via DropletUtils [47]) were removed for quality control. Then, 25 normal BMMC samples were selected for cell-type annotation and normal reference construction. The expression profiles of 103,871 cells from the 25 samples were merged as input for the Seurat analysis. The Seurat package, with default settings, was used to normalize and scale the expression matrix. To reduce the dimensionality of the expression matrix, a principal component analysis was performed on 3000 highly variable genes. Batch effects were adjusted using Harmony [26] with parameters set to max.iter.harmony = 5 and sigma = 0.15. Unsupervised cell clusters were acquired using a graph-based clustering approach (Louvain algorithm, top 30 dimensions selected, resolution = 1), and visualized via Uniform Manifold Approximation and Projection (UMAP) [48] with dimensionality reduction. The single-cell source expression data of 25 normal BMMC samples for HematoMap are accessible on figshare (https://doi.org/10.6084/m9.figshare.24447412).

### Annotation of cell types in normal BMMCs

The cell-type annotation of normal BMMCs was conducted using the following steps: (1) Identification of major cell populations. Unsupervised clustering was performed on the normal BMMCs, and 38 clusters were identified. Marker genes of each cluster were calculated using the “FindAllMarkers” function with the following criteria: log_2_ (fold change) > 0.25, min.pct > 0.1, and adjusted *P* value < 0.05. The cells were annotated using both the machine-learning-based software SingleR [30] and the high expression of canonical hematopoietic markers (*i.e.*, *CD34* for HSPCs, *CD14* for monocytes, *CD1C* for DCs, *CD3* for T cells, *CD79A* for B cells, and *CA1* for erythrocytes) in each cluster. Four lineages (HSPCs, myeloid cells, B cells, and T/NK cells), including eight cell populations (HSPCs, monocytes, DCs, erythrocytes, B cells, CD8^+^ T cells, CD4^+^ T cells, and NK cells), were identified. (2) Identification of cell types. Cells in each population were extracted and analyzed via an independent Seurat workflow. For example, to identify cell types in HSPCs, the expression matrix for HSPCs was normalized and scaled, and unsupervised cell clusters were acquired using a graph-based clustering approach. Cell markers for each cluster were calculated. The Max-Min *K*-means algorithm was used to generate subclusters. The cell types were identified by the relative expression levels of canonical hematopoietic markers (Table S2). (3) Confirmation of cell types. The cell marker expression levels of 1079 subclusters were confirmed, and the cell-type annotations were rechecked manually.

### Inference of lineage aberrations using scRNA-seq

The stages of cell differentiation form a classical hematopoietic hierarchy [49,50]. HSCs/MPPs are at the apex of the hematopoietic hierarchy and produce precursors of various blood cell lineages, such as megakaryocytes, erythrocytes, monocytes, granulocytes, B cells, and T/NK cells. We used hierarchy-based visualization to visualize normal hematopoiesis. To estimate the similarities between the normal and leukemic subclusters, we first calculated their cosine distances. The LIKE score of each leukemic subcluster was determined using the cosine distance to the normal subcluster and the distance to the differentiation process initiating from the HSC/MPP. Lineage aberrancy was estimated based on the ratio of the percentage of leukemic BMMCs to that of normal BMMCs.

The cosine similarity distance was used to quantify the similarities between the gene expression profiles of leukemia-associated subclusters (matrix A) and normal BMMCs and HSC/MPP subclusters (matrix B). The cosine similarity, symbolized as *sim*(A, B), is calculated by taking the dot product of matrices A and B and dividing this by the product of their magnitudes:


$$\mathrm{sim}\left(A,\;B\right)=\frac{A\cdot B}{\left|\left|A\right|\right|\left|\left|B\right|\right|}\;$$

Subsequently, *d*(A, B) was defined as the distance between subclusters as one minus the cosine similarity:


$$d\left(A,\;B\right)=1-\mathrm{sim}\left(A,\;B\right)$$

The LIKE score for each leukemia-related subcluster was then derived on the basis of these distances. For most cell types, this score is a product of the similarity score between the leukemia subcluster and the normal BMMC subcluster, adjusted by the similarity score to the differentiation process initiating from the HSC/MPP. However, for T/NK cells — a category distinct in its lineage — the LIKE score is equated directly to the similarity score with its normal counterpart:


$$\mathrm{like}\left(A,\;B\right)=\left\{\begin{array}{c} sim\left(A,\;B\right)*\;\text{sin}\;\left(\theta \right)\;(\mathrm{Others})\\ sim\left(A,\;B\right)\;(T/\mathrm{NK}\;\mathrm{cells}) \end{array}\right.$$

### RNA-seq, alignment, and expression analyses

Raw FASTQ files were aligned to the human reference genome. For gene expression analyses, the human reference genome GRCh38 (v40) and its annotation file were downloaded from the GENCODE database (https://www.gencodegenes.org/). Salmon (v1.8.0) [51] was used to generate the counts and the transcripts per kilobase of exon model per million mapped reads matrix for all the patients. For the BCP-ALL and AML cohorts from multiple centers, batch effects were adjusted using the sva package [52]. For comparisons with normal BMMCs, the “normalizeBetweenArrays” function in the limma package [53] was used to normalize expression among BCP-ALL, AML, and normal BMMCs.

### Establishment of a LASSO-based score model

The mean expression values of the 1079 subclusters were used for the LASSO regression analysis, and a 10-fold cross-validation was applied to determine the optimal value of penalty parameter *λ* with the R package glmnet [54]. Subclusters were separated by the related cell types. A LASSO model was constructed independently for each cell type.

## Code availability

The source code for the R package is available on GitHub (https://github.com/NRCTM-bioinfo/HematoMap). The source code has also been submitted to BioCode at the NGDC, CNCB (BioCode: BT007581), which is publicly accessible at https://ngdc.cncb.ac.cn/biocode/tool/7581. For a detailed tutorial on HematoMap, please visit our online tutorial at https://nrctm-bioinfo.github.io/HematoMap/index.html.

## Supplementary Material

qzaf005_Supplementary_Data

## CRediT author statement

**Yuting Dai:** Methodology, Software, Writing − original draft, Visualization, Validation, Funding acquisition. **Wen Ouyang:** Methodology, Software, Writing − original draft, Visualization, Data curation. **Wen Jin:** Resources, Validation. **Fan Zhang:** Resources, Validation, Funding acquisition. **Wenyan Cheng:** Resources. **Jianfeng Li:** Resources. **Shuo He:** Validation, Visualization. **Junqi Zong:** Validation, Visualization. **Shijia Cao:** Validation, Visualization. **Chenxin Zhou:** Validation, Visualization. **Junchen Luo:** Validation, Visualization. **Gang Lv:** Resources. **Jinyan Huang:** Resources. **Hai Fang:** Supervision, Funding acquisition, Writing − review & editing. **Xiaojian Sun:** Supervision, Writing − review & editing. **Kankan Wang:** Supervision, Funding acquisition, Writing − review & editing. **Saijuan Chen:** Supervision, Writing − review & editing. All authors have read and approved the final manuscript.

## ORCID

0000-0001-8275-2115 (Yuting Dai)

0009-0009-1962-9099 (Wen Ouyang)

0000-0002-5084-2076 (Wen Jin)

0000-0001-5229-7529 (Fan Zhang)

0000-0003-4347-5981 (Wenyan Cheng)

0000-0003-2349-208X (Jianfeng Li)

0009-0009-0941-4908 (Shuo He)

0009-0006-4882-6858 (Junqi Zong)

0009-0004-3757-2341 (Shijia Cao)

0009-0007-3921-2536 (Chenxin Zhou)

0009-0009-5108-9854 (Junchen Luo)

0000-0003-2573-8813 (Gang Lv)

0000-0002-8053-0209 (Jinyan Huang)

0000-0003-3961-8572 (Hai Fang)

0000-0001-8826-4614 (Xiaojian Sun)

0000-0001-7198-2134 (Kankan Wang)

0000-0003-3789-1284 (Saijuan Chen)
