# Supplementary material for: Resolving Leukemia Heterogeneity and Lineage Aberrations with HematoMap
Source: Genomics Proteomics Bioinformatics. 2025 Feb 13;23(2):qzaf005. doi: 10.1093/gpbjnl/qzaf005 (PMC12343003; doi:10.1093/gpbjnl/qzaf005)
Supplement: qzaf005_Supplementary_Data [file qzaf005_supplementary_data.zip › FigureS3.pdf]

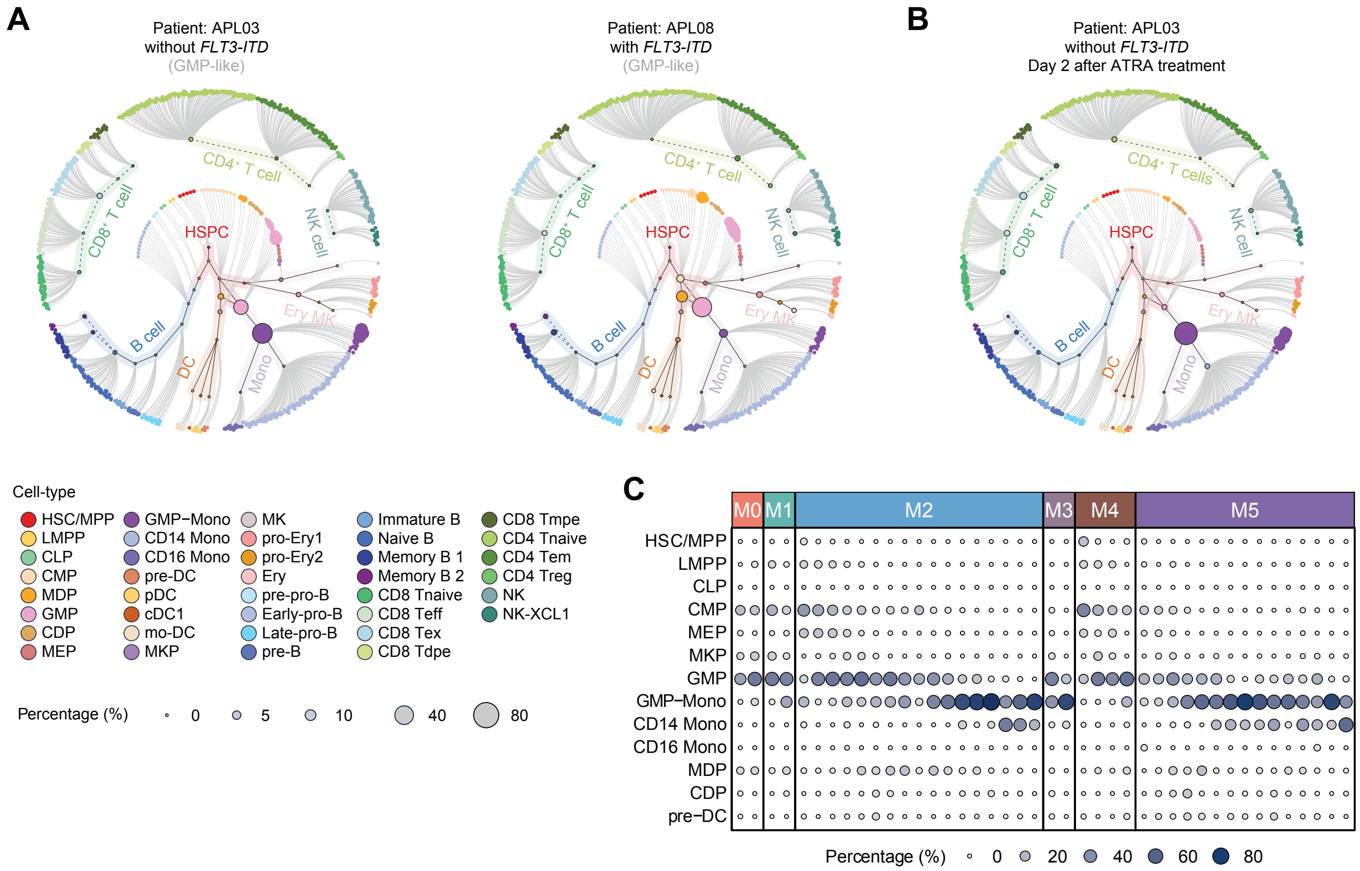
A
Patient: APL03
without FLT3-ITD
(GMP-like)
Patient: APL08
with FLT3-ITD
(GMP-like)
B
Patient: APL03
without FLT3-ITD
Day 2 after ATRA treatment
CD4+ T cell
CD4+ T cells
CD8+ T cell
NK cell
HSPC
Ery MK
B cell
DC
Mono
Cell-type
HSC/MPP
LMPP
CLP
CMP
MDP
GMP
CDP
MEP
GMP−Mono
CD14 Mono
CD16 Mono
pre-DC
pDC
cDC1
mo-DC
MKP
MK
pro-Ery1
pro-Ery2
Ery
pre-pro-B
Early-pro-B
Late-pro-B
pre-B
Immature B
Naive B
Memory B 1
Memory B 2
CD8 Tnaive
CD8 Teff
CD8 Tex
CD8 Tdpe
CD8 Tmpe
CD4 Tnaive
CD4 Tem
CD4 Treg
NK
NK-XCL1
Percentage (%) 0 5 10 40 80
C
M0
M1
M2
M3
M4
M5
HSC/MPP
LMPP
CLP
CMP
MEP
MKP
GMP
GMP−Mono
CD14 Mono
CD16 Mono
MDP
CDP
pre−DC
Percentage (%) 0 20 40 60 80
