# Supplementary material for: Resolving Leukemia Heterogeneity and Lineage Aberrations with HematoMap
Source: Genomics Proteomics Bioinformatics. 2025 Feb 13;23(2):qzaf005. doi: 10.1093/gpbjnl/qzaf005 (PMC12343003; doi:10.1093/gpbjnl/qzaf005)
Supplement: qzaf005_Supplementary_Data [file qzaf005_supplementary_data.zip › Supplementary material captions.docx]

## Supplementary material

**Figure S1 Characterization of the cellular composition of normal BMMCs**

**A.** UMAP visualization of major cell populations in normal BMMCs. Eight populations were identified, including eight hematopoiesis/immune-related populations. **B.** Violin plot of classical marker expression of the eight hematopoiesis/immune-related populations. **C.** Distributions of the cell numbers of the 1079 subclusters identified in normal BMMCs. **D.** DPT of the 1079 subclusters. **E.** Boxplot of the DPT in different cell types. **F.** Bar plot of the percentages of 25 normal BMMCs. **G.** Dot plot of the robustness evaluation using different sample sizes. Spearman’s correlation between the expression profiles of the reference and the downsampled subclusters within different sample numbers. Each dot represents one test, and each test was repeated 20 times using different random seeds. When the sample size reached 12, the mean Spearman’s correlation coefficient exceeded 0.9, indicating a high correlation. When the sample size reached 17, the coefficient exceeded 0.95, indicating a very strong correlation. **H.** Dot plot of the robustness evaluation using different cell numbers. **I.** Annotation accuracy of annotations from external 10X Genomics data of normal BMMCs. The subclusters obtained from the 25 normal BMMCs were input as the reference. The annotation accuracy of each cell population was also illustrated using a boxplot. **J.** ROC curve and AUC analyses revealed favorable performance when the annotation reference constructed from the external normal BMMCs of 10X Genomics was used. **K.** Boxplot illustrating the annotation accuracy comparing annotations using external non-10X Genomics data of normal BMMCs. **L.** ROC curves and AUCs using external normal BMMCs from non-10X Genomics. DPT, diffusion pseudotime.

**Figure S2 Calculation of similarities between subclusters in APL and normal BMMCs**

**A.** Visualization of the theta values and the LIKE scores. The upper panel shows the theta values and LIKE scores of the four selected subclusters. The maximum LIKE scores of the four subclusters correspond to the GMP, GMP-monocyte, naive B cell, and CD4 Tem. The lower panel shows the hierarchy-based circular plot of the LIKE score. The edge of the circle illustrates the LIKE score of normal subclusters. The inner circle has the highest LIKE score value. Thus, for one subcluster in APL, the theta value and the LIKE score were calculated, and the highest LIKE score was chosen to annotate these subclusters. **B.** Violin plot of the expression values of GMP/GMP-monocyte-related markers in the APL samples. The cells were annotated on the basis of the LIKE score. **C.** Hierarchy-based visualization of the cellular composition of *de novo* APL and normal BMMCs. The dots are colored according to their relative proportions. **D.** Comparison of the LIKE scores calculated from normal BMMCs and APLs. **E.** ROC curve illustrating the favorable performance of using the LIKE score to annotate the 25 normal BMMCs. **F.** Hierarchy-based visualization of the cellular composition of two additional healthy BMMCs. The dots are colored according to their relative proportions. Normal BMMCs were Young-1 (31-year-old female) and Aged-1 (76-year-old male). **G.** Tree plot visualization of the lineages of the two additional normal BMMCs. The cell types, represented as circles, are color-coded, with HSC/MPP sitting at the initiation site of the hierarchy. The size of each circle represents the relative ratio of cell proportions compared with the normal reference built into HematoMap. A relative ratio greater than 1 indicates proliferation, whereas a ratio less than 1 indicates suppression. The solid lines depict continuous differentiation processes within bone marrow hematopoiesis, whereas the dashed lines indicate cells recruited into the bone marrow from other tissues, such as T and NK cells.

**Figure S3 Inference of lineage aberrancy in AML scRNA-seq cohorts and patients with different treatment time points**

**A and B.** Hierarchy-based visualization of the *de novo* AML patients (**A**) and APL patients after two days of ATRA treatment (**B**). **C.** Cellular compositions of the HSPCs and myeloid lineages of the 40 *de novo* AML patients in scDS5 and two APL patients. Each column represents one patient, and the dot is sized by the inferred cellular percentages. Patients were ordered by the FAB classification. M0: undifferentiated acute myeloblastic leukemia; M1: acute myeloblastic leukemia with minimal maturation; M2: acute myeloblastic leukemia with maturation; M3: APL; M4: acute myelomonocytic leukemia; M5: acute monocytic leukemia. FAB, French–American–British.

**Figure S4 Inference of cellular abundance changes in the *de novo* AML and BCP-ALL bulk cohorts**

**A.** Boxplot of the normalized LASSO score of the 38 cell types in normal BMMCs from bulk RNA-seq. Dots represent samples. The two dotted lines represent −0.32 and 0.32, which means that the fold changes were set to 0.8 and 1.25 (log_2_-transformed fold changes of −0.32 and 0.32, respectively). Using this cutoff, 95% of the dots were included between the two cutoffs. LASSO scores were normalized by subtracting the mean value of normal BMMCs. **B.** Heatmap and lollipop plot visualization of the normalized LASSO score in AML patients. The mean values of each AML subgroup were calculated and visualized using a lollipop plot. *P* values were calculated via ANOVA and adjusted according to the FDR. The LASSO score was normalized by subtracting the mean value of the LASSO score obtained from normal BMMCs. **C.** Heatmap and lollipop plot visualization of the normalized LASSO score in BCP-ALL patients. The mean values of each BCP-ALL subgroup were calculated and visualized using a lollipop plot. **D.** Lollipop plot visualization of the mean normalized LASSO score in AML FAB subtypes. **E.** Lollipop plot visualization of the mean normalized LASSO score in the TCGA-LAML FAB subtypes. **F.** Lollipop plot visualization of the mean normalized LASSO score in Beat-AML FAB subtypes. TCGA, The Cancer Genome Atlas; LAML, acute myeloid leukemia-like; FDR, false discovery rate.

**Table S1 Overview of data collection utilized in this study**

**Table S2 Structure of hierarchy-based cell types in BMMCs and major gene markers of each cell type**

**Table S3 Sample information and cellular compositions of normal BMMCs**

**Table S4 Coefficients of the LASSO score model of the 38 cell types**

**Table S5 Cell-type-based LASSO score of normal BMMCs**
