# Supplementary figures and images for: Resolving Leukemia Heterogeneity and Lineage Aberrations with HematoMap
Source: Genomics Proteomics Bioinformatics. 2025 Feb 13;23(2):qzaf005. doi: 10.1093/gpbjnl/qzaf005 (PMC12343003; doi:10.1093/gpbjnl/qzaf005)

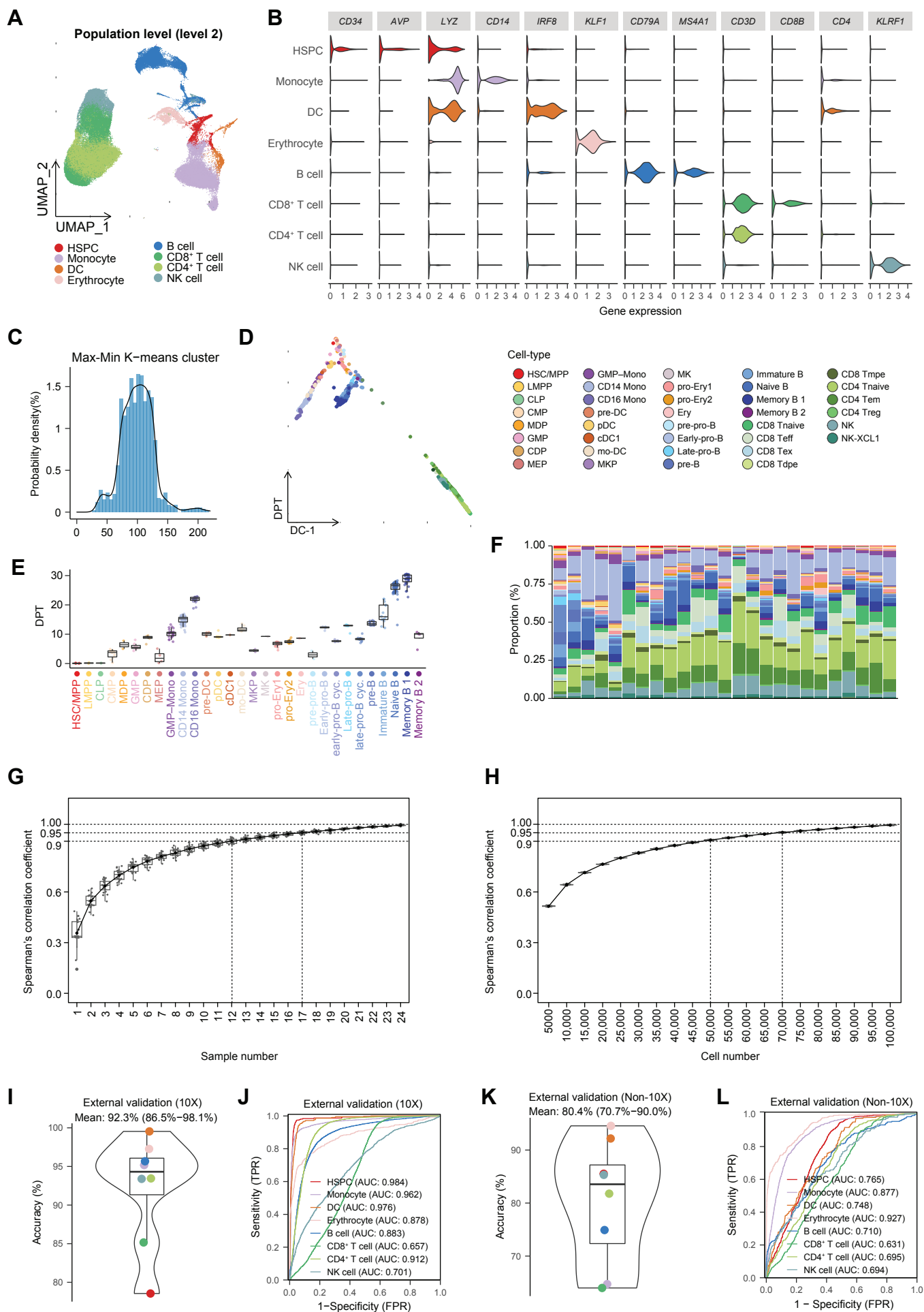

Supplement: qzaf005_Supplementary_Data [file qzaf005_supplementary_data.zip › FigureS1.pdf]

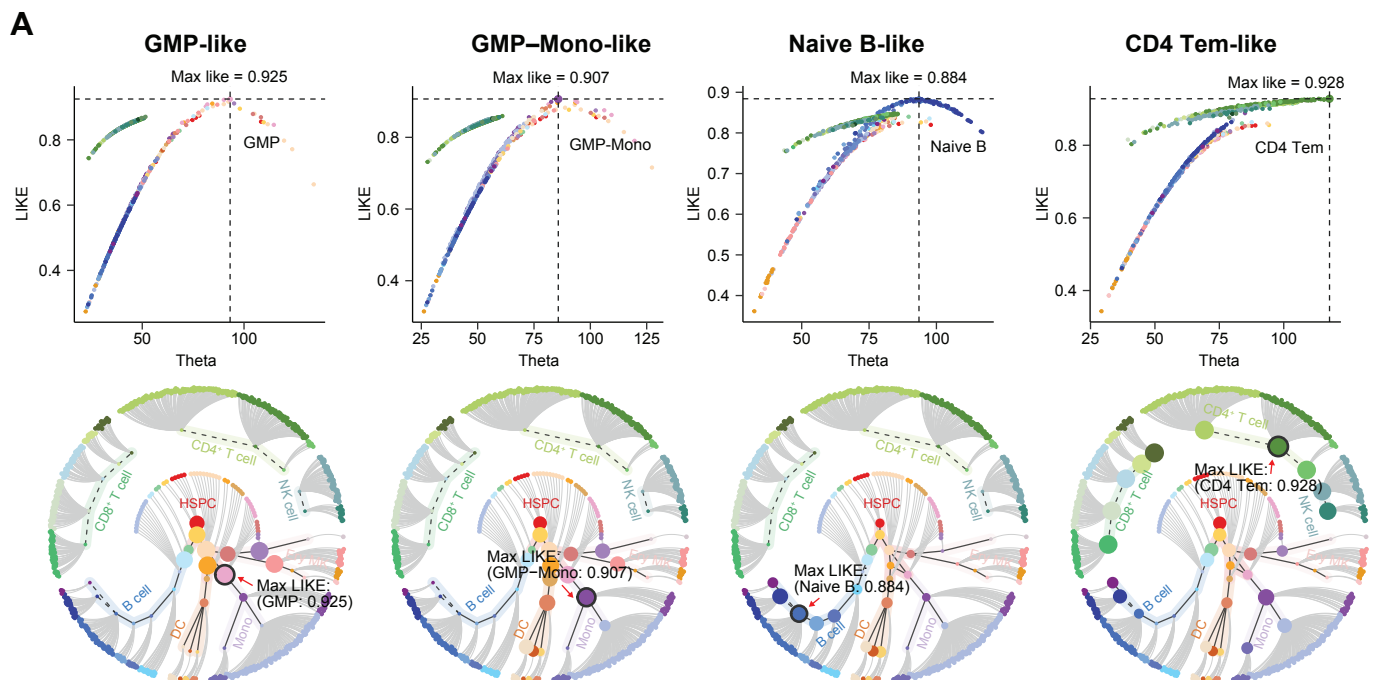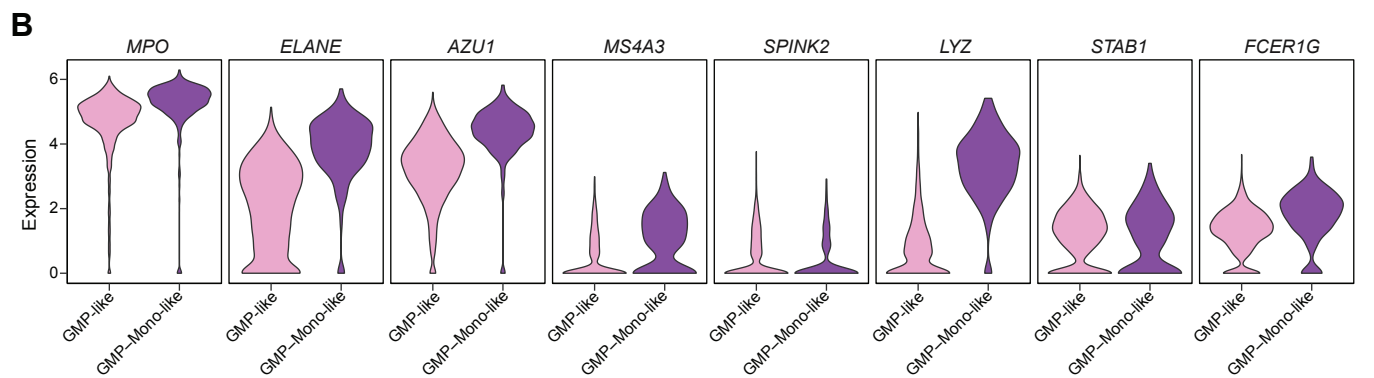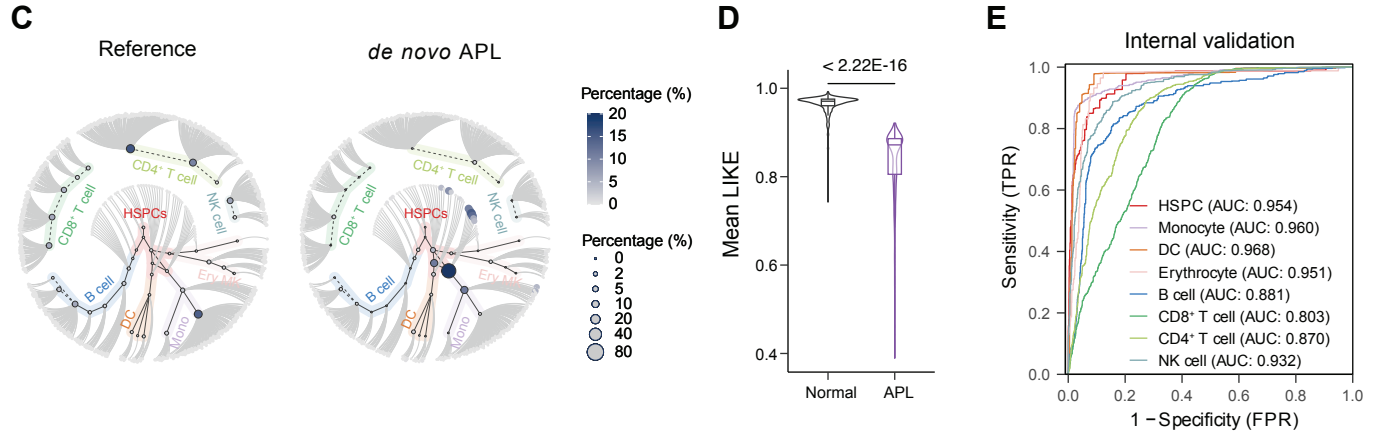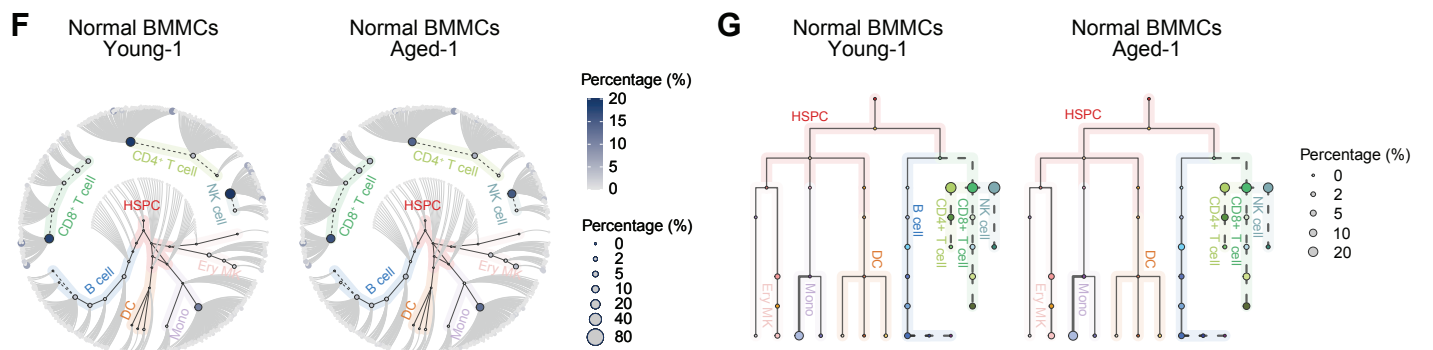

Supplement: qzaf005_Supplementary_Data [file qzaf005_supplementary_data.zip › FigureS2.pdf]

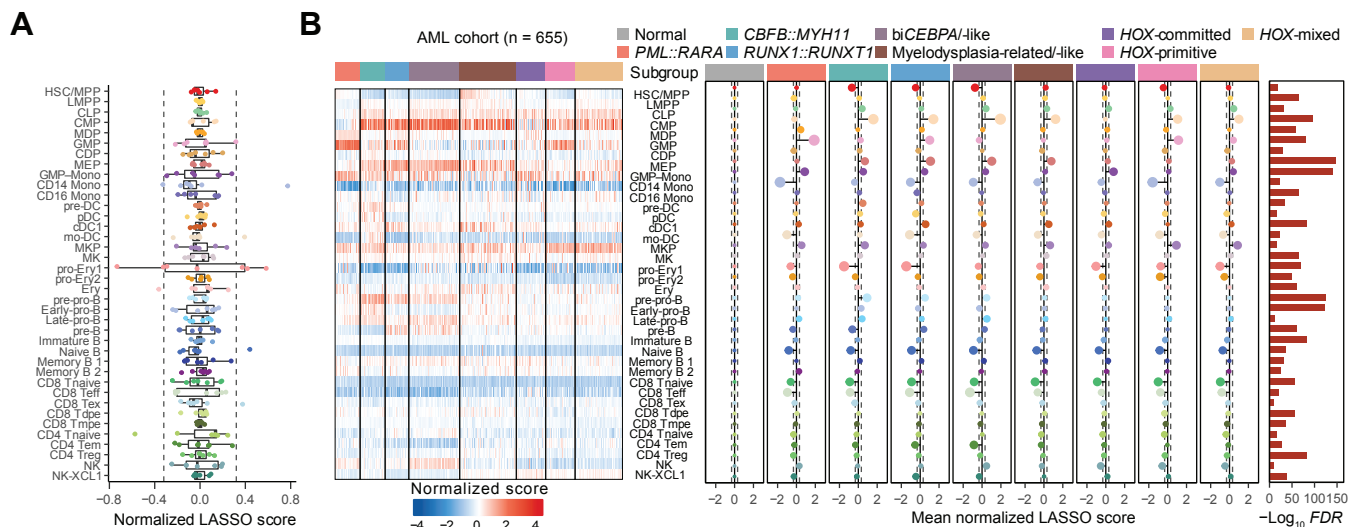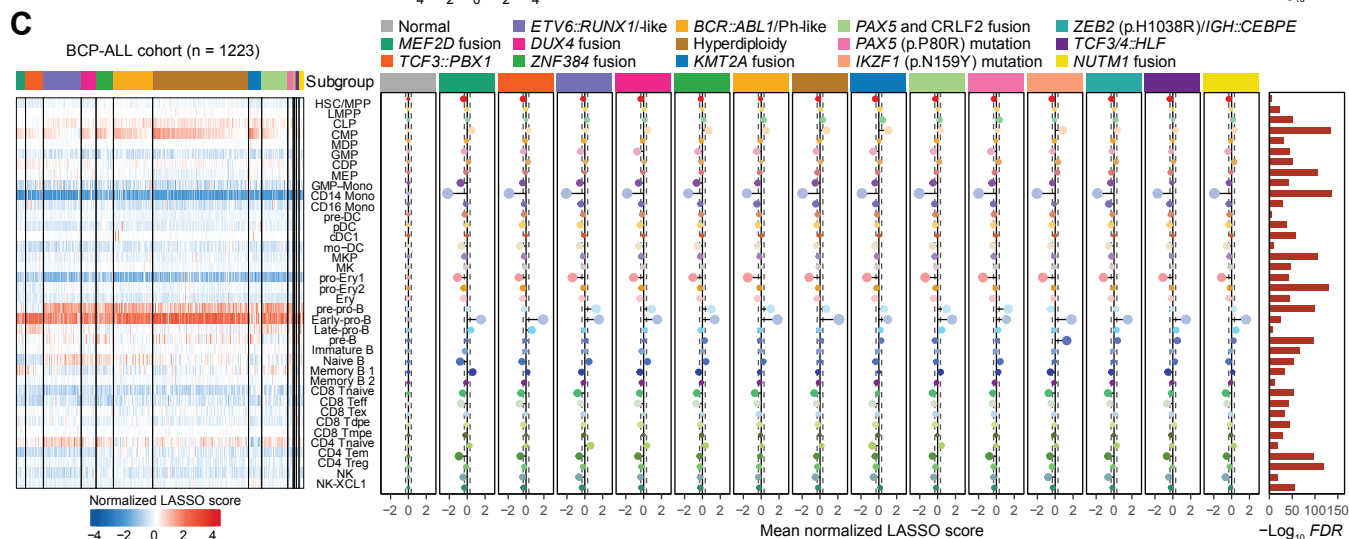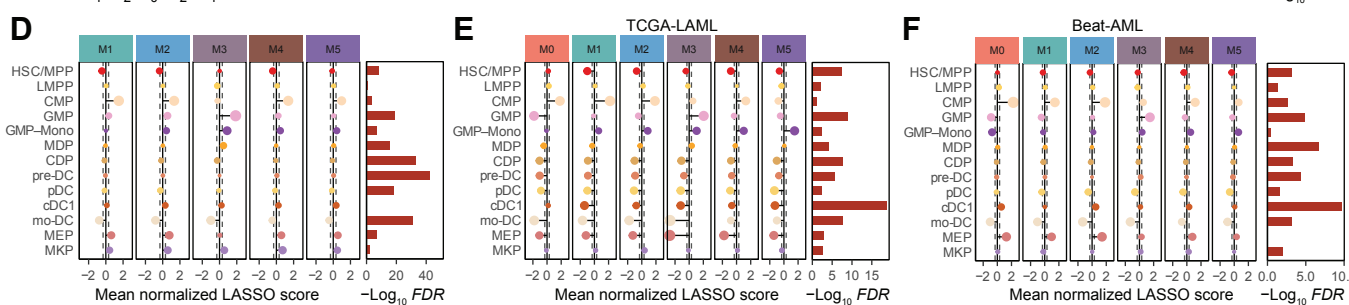

Supplement: qzaf005_Supplementary_Data [file qzaf005_supplementary_data.zip › FigureS4.pdf]
